# Papez’s Forgotten Tract: 80 Years of Unreconciled Findings Concerning the Thalamocingulate Tract

**DOI:** 10.3389/fnana.2019.00014

**Published:** 2019-02-18

**Authors:** Joshua Weininger, Elena Roman, Paul Tierney, Denis Barry, Hugh Gallagher, Paul Murphy, Kirk J. Levins, Veronica O’Keane, Erik O’Hanlon, Darren W. Roddy

**Affiliations:** ^1^REDEEM Group, Department of Psychiatry, Trinity College Dublin, Dublin, Ireland; ^2^Department of Anatomy, Trinity College Dublin, Dublin, Ireland; ^3^Department of Anaesthesia, Intensive Care and Pain Medicine, St. Vincent’s University Hospital, Dublin, Ireland; ^4^Department of Physiology, School of Medicine, University College Dublin, Dublin, Ireland

**Keywords:** thalamocingulate tract, thalamus, cingulum, Papez circuit, cingulate cortex, anterior thalamic radiation, anterior thalamic nuclei

## Abstract

The thalamocingulate tract is a key component of the Papez circuit that connects the anterior thalamic nucleus (ATN) to the cingulum bundle. While the other white matter connections, consisting of the fornix, cingulum bundle and mammillothalamic tract, were well defined in Papez’s original 1937 paper, the anatomy of the thalamocingulate pathway was mentioned only in passing. Subsequent research has been unable to clarify the precise anatomical trajectory of this tract. In particular, the site of thalamocingulate tract interactions with the cingulum bundle have been inconsistently reported. This review aims to synthesize research on this least studied component of the Papez circuit. A systemic approach to reviewing historical anatomical dissection and neuronal tracing studies as well as contemporary diffusion magnetic resonance imaging studies of the thalamocingulate tract was undertaken across species. We found that although inconsistent, prior research broadly encompasses two differing descriptions of how the ATN interfaces with the cingulum after passing laterally through the anterior limb of the internal capsule. The first group of studies show that the pathway turns medially and rostrally and passes to the anterior cingulate region (Brodmann areas 24, 33, and 32) only. A second group suggests that the thalamocingulate tract interfaces with both the anterior and posterior cingulate (Brodmann areas 23 and 31) and retrosplenial region (Brodmann area 29). We discuss potential reasons for these discrepancies such as altering methodologies and species differences. We also discuss how these inconsistencies may be resolved in further research with refinements of terminology for the cingulate cortex and the thalamocingulate tract. Understanding the precise anatomical course of the last remaining unresolved final white matter tract in the Papez circuit may facilitate accurate investigation of the role of the complete Papez circuit in emotion and memory.

## Introduction

**FIGURE 1:**
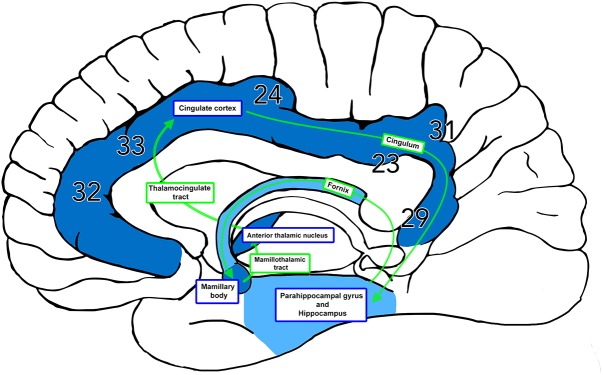
The Papez Circuit. The thalamocingulate tract connects the anterior thalamic nuclei through the cingulate cortex in the cingulum, then travels in a circuit through the parahippocampal gyrus and hippocampus, fornix, mammillary bodies, and mammillothalamic tract back to the anterior cingulate gyrus. Brodmann areas (BA) 32, 33, and 24 correspond to the anterior cingulate cortex. BA 23 and 31 correspond to the posterior cingulate cortex. BA 29 corresponds to the restrosplenial cortex.

The Papez circuit was described in 1937 by James Wenceslaus Papez in his paper “A Proposed Mechanism of Emotion” (Papez, 1937). This pioneered a new understanding of how the limbic lobe directed emotions and memory through its connections with the hypothalamus. Papez presented a description of the hippocampus, hypothalamus (mammillary bodies), anterior thalamus, cingulate gyrus and their interconnections (Figure 1) as the “anatomic basis of the emotions.” Research over the following 80 years has consolidated our understanding of the central role of this circuit in limbic activities, although it has subsequently emerged as more important in the function of memory than in emotional processing (LeDoux, 1993; Aggleton and Brown, 1999). Anatomical dissection-based studies and neuroimaging techniques have consistently described the precise white matter interconnections between circuit hubs including the cingulum (Shah et al., 2012; Jones et al., 2013), perforant pathway (Augustinack et al., 2010; Zeineh et al., 2017), fornix (Carpenter, 1985; Metzler-Baddeley et al., 2013), and mammillothalamic tract (Tubbs et al., 2011; Kamali et al., 2018). Although an integral component of the Papez circuit, the thalamocingulate tract connecting the anterior thalamic nuclei to the cingulum bundle is less consistently described in both the anatomical and neuroimaging literature.

### Anterior Thalamic Nucleus

The anterior thalamic nucleus (ATN) consists of a group of subnuclei within the rostrodorsal region of the thalamus and is a key component of the hippocampal system for episodic memory (Child and Benarroch, 2013). It is separated from the rest of the thalamus by the Y-shaped anterior internal medullary lamina (Jones, 2007). Classically it can be subdivided into three subnuclei, the anteromedial (AM), anterodorsal (AD) and anteroventral (AV) nuclei (Jones, 2007; Mai and Paxinos, 2011). Adjacent to the anterior thalamic nuclei lies the laterodorsal (LD) nucleus which has been described as being closely related in terms of connectivity (Schmahmann and Pandya, 2006). The key inputs through the Papez circuit to the anterior thalamic nuclei originate from the main hippocampal output region of the subicular regions (including pre and parasubiculum), with lesser contributions from hippocampal cornu ammonis one (CA1) (Child and Benarroch, 2013). These inputs reach the anterior thalamus either directly through the fornix or indirectly via the mamillary body and mammillothalamic tract (Aggleton, 2008). Clinically this nucleus has a role in episodic memory, as it and its afferent connections implicated in anterograde memory deficits, e.g., in Korsakoff’s syndrome (Kril and Harper, 2012). Additionally, the nucleus may be affected by prion diseases such as fatal familial insomnia (Manetto et al., 1992) and Creutzfeldt-Jakob disease (Tschampa et al., 2002). The anterior thalamus may also have a role in seizure propagation, as demonstrated by the success of deep brain stimulation of the nucleus in patients with refractory epilepsy (Lega et al., 2010). Through its connections with the anterior cingulate and prefrontal regions, the ATN may also contribute to hippocampal-prefrontal interactions involved in emotional and executive functions and as such may be involved in mood disorders such as depression (Sun et al., 2015).

### Cingulum

The cingulum is a distinctive white matter tract that forms a near-complete ring underneath the cingulate gyrus from the orbitofrontal cortex anteriorly to the temporal pole posteriorly (Jones et al., 2013; Wu et al., 2016). It consists of both long and short association fibers along its length, connecting cortical and subcortical regions (Yakolev et al., 1961; Schmahmann and Pandya, 2006; Heilbronner and Haber, 2014). The sizeable connectivity of the cingulum facilitates its involvement in diverse cortical processes such as executive function, attention, emotional and behavioral regulation, memory and visuospatial processing (Bubb et al., 2018). Such activity may be location dependent within the cingulum itself reflecting its inherent microstructural connectivity and the properties of the overlying cingulate cortex, with the anterior cingulum potentially having more involvement in emotional regulation and the posterior cingulum and retrosplenial regions having more involvement in cognitive control (Wu et al., 2016). The cingulum bundle has been implicated in many neuropsychiatric diseases including schizophrenia (Whitford et al., 2014), depression (Bhatia et al., 2018), post-traumatic stress disorder (Sanjuan et al., 2013), obsessive compulsive disorder (Gruner et al., 2012), autism (Shukla et al., 2011) and Alzheimer’s disease (Catheline et al., 2010; Bubb et al., 2018).

### Thalamocingulate Tract and Thalamic Radiations

Connections between the ATN and the cingulum were first described in the rat brain by Clark and Boggon (1933) and Waller (1934). Though the termination of fibers in various portions of the cingulum was described in some detail, the anatomical pathway of the tract as it makes its way from diencephalon to telencephalon was not clarified. Shortly afterward Papez described his emotional circuit with the connection between the anterior thalamus and cingulum as a fundamental component. Again, discussion is given to the “connections” or terminations of these fibers within the cingulum, but the precise anatomical course is not outlined;

“*The course of these fibers has not been illustrated or described in detail. In general, they pass laterally and forward from the anterior and anterodorsal nuclei to enter the anterior limb of the internal capsule. They turn dorsally and medially over the anterior horn of the ventricle to enter the cingulum in the medial wall of the hemisphere.*”

It is also unclear what species Papez is referring to, having studied “various mammals (e.g., dog, monkey and man).” The fibers were described rather generally as reaching their destination “by the medial thalamocortical radiation,” a non-defined entity. It is most likely he was suggesting that the thalamocingulate tract travels through the anterior thalamic radiation (otherwise known as the anterior thalamic peduncle). The anterior thalamic radiation is a wide fan-shaped tract that connects the thalamic anterior and mediodorsal nuclear groups to the frontal lobe and anterior cingulate cortex (Carpenter, 1985; Nieuwenhuys et al., 2007). It traverses the anterior limb of the internal capsule, but the thalamocingulate component of the radiation has not been well described. In particular, the precise location where the thalamocingulate tract fuses with the cingulum remains ill-defined with disagreement in the literature in describing the course of the anterior thalamic radiations entering the cingulate cortex and cingulum. The interfacing portion of the cingulate cortex has been a primary point of controversy, and secondarily how the fibers from the anteroventral, anteromedial, and anterodorsal thalamic nuclei diverge from one another.

Defining the course of the thalamocingulate tract is necessary for certain types of neuroimaging designed to investigate white matter integrity. Diffusion-weighted imaging (DTI) allows tracts to be reconstructed by analyzing the Brownian motion of water molecules as they move within axonal bundles (Mori and Aggarwal, 2014). Using complex algorithms, white matter streamlines throughout the brain can be generated. However, interpretation of the streamlines into individual tracts needs considerable understanding of the complex anatomy of the particular region of interest to prevent misreading and subsequent mislabeling of the structures. Correspondingly, how the ATN relates to the cingulum through either the anterior or posterior cingulate gyrus has relevance to future imaging of the Papez circuit in emotional and memory processing. The posterior cingulate gyrus has been implicated in the functioning of the default mode network, de-activating when attention is externally directed, while the anterior cingulate gyrus through dorsal connections with frontal and parietal lobes and ventral connections to limbic structures contributes to a diversity of cognitive, emotional, and motor functions (Schmahmann and Pandya, 2006).

The anatomical course of the thalamocingulate tract remains uncertain despite 80 years since Papez’s original work. Clarifying the precise course of the tract will aid future anatomical and diffusion-weighted tractography studies into the function of the Papez circuit, allowing greater insights into its role in neuropsychiatric disorders. In order to address the uncertainty concerning the pathway of the tract, a systematic review of the anatomical course of the thalamocingulate tract was undertaken.

## Methods

This literature review was conducted using PubMed/MEDLINE, Google Scholar, EMBASE, The Cochrane Library, OVID and PsycINFO. Studies were identified with the keywords “thalamocingulate tract,” “anterior thalamic projections,” “thalamocortical radiations,” “anterior thalamic nuclei to cingulate cortex,” and “Papez circuit.” Resources such as older texts within the Anatomy Department of Trinity College Dublin and Trinity College Library were also consulted. References in all studies were checked and appropriate studies were identified. Studies that described the thalamocingulate tract using anatomical dissections, neuronal tracing techniques or neuroimaging were included. Information pertaining to the course of the tract was examined with respect to methods, genus and the specific termination of the thalamocingulate fiber tracts.

## Results

**FIGURE 2:**
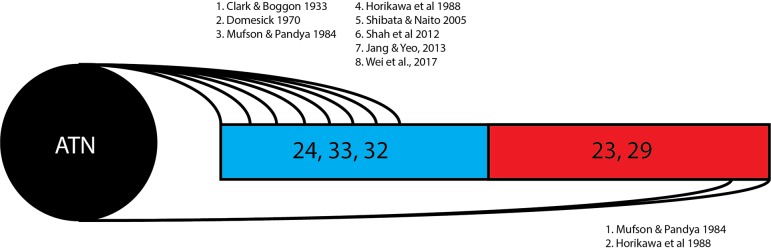
Thalamocingulate tract entry into cingulum. Diagrammatic overview of the quantity of studies showing thalamic connections to each cingular region. Brodmann area 24, 33, and 32 (shown in blue) correspond to the anterior cingulate cortex. Brodmann area 23 and 29 (shown in red) correspond to the posterior cingulate cortex and retrosplenial cortex. ATN, anterior thalamic nucleus.

**FIGURE 3:**
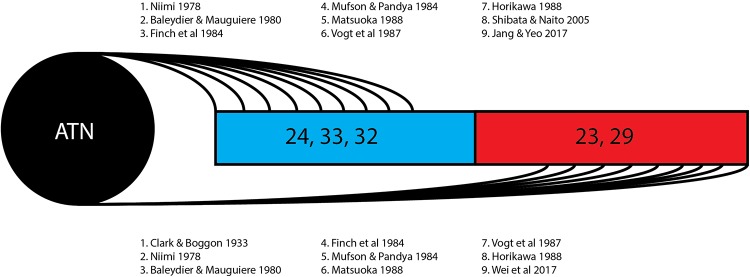
Thalamocingulate tract termination in cingulum. Diagrammatic overview of the quantity of studies showing regions of thalamic termination once enclosed within the cingulum bundle. Brodmann area 24, 33, and 32 (shown in blue) correspond to the anterior cingulate cortex. Brodmann area 23 and 29 (shown in red) correspond to the posterior cingulate cortex and retrosplenial cortex. ATN, anterior thalamic nucleus.

This review of 13 frequently cited studies (summarized in Table 1) and three case reports identifies two broad hypotheses of how the thalamocingulate tract projects between the anterior thalamic nuclei and the cingulate region. These groupings (shown in Figure 2) either portray the white matter projecting exclusively anteriorly into the anterior cingulate cortex (ACC) or as having additional projections caudally into the posterior cingulate cortex (PCC) and retrosplenial cortex (RSC). The ACC corresponds to Brodmann Areas (BA) 32, 33, and 24, while the PCC and RSC corresponds to BA 23, 29, and 31 (Figure 1). Additionally, another group of studies which were cited in the literature as establishing the course of the tract were found to not provide this information but rather only the points of termination in the cingulate cortex (Figure 3). These studies have implications in supporting and excluding anatomical hypotheses and have functional relevance despite not determining the course.

**Table 1:** The literature review findings are shown.

Author(s)	Year	Method	Subjects	Cingular Entry	Cingular Termination	Notes
Clark and Boggon	1933	Lesion-induced degradation tracing, cat and rat	4 rats, 3 cats	ACC	PCC/RSC	Rostral projection from the ATN to the level of the rostral corpus callosum, then curving medially to join into the dorsal cingulate gyrus.
Domesick et al.	1970	Lesion-induced axon degradation tracing	15 rats	ACC	*None described*	ATN fibers project rostrally and dorsally to the ACC. Once enclosed in the cingulum the fibers pass caudally.
Niimi et al.	1978	Electrolytic lesion-induced degeneration tracing	13 cats	*None described*	ACC and PCC/RSC	AD terminates at BA 29 AV terminates at BA 23 and 29 AM terminates at BA 24
Baleydier and Mauguiere	1980	Horseradish peroxidase (HRP) axonal tracing	13 monkeys, multiple species	*None Described*	ACC and PCC/RSC	*Efferent* AV, AD, LD terminate in BA 23 AM terminates in BA 24 *Afferent* BA 23 projects to AV, AM, LD BA 24 projects to AM
Finch et al.	1984	HRP axonal tracing	75 rats	*None described*	ACC and PSC/RSC	AD terminates in BA 23 AV terminates in BA 29 AM had a lack of significant cingular termination
Mufson and Pandya	1984	Radiolabelled amino acids	16 Rhesus monkeys	ACC and PCC/RSC	ACC and PCC/RSC	ATN projects rostrally beneath caudate nucleus, through anterior limb of internal capsule, spreads in a laminar fan-like projection to the ventral sector of cingulum, spanning all areas (BA 24, 25, 32, 23, 29, 30).
Matsuoka	1986	HRP axonal tracing	8 cats	*None described*	ACC and PCC/RSC	Rostral cingulate gyrus receives fibers from the AM and part of the AV. Caudal part of cingulate gyrus receives fibers from the AM and AV. Intermediate cingulate gyrus receives fibers from parts of AM and AV. None from AD.
Vogt et al.	1987	HRP axonal tracing	15 rhesus monkeys	*None described*	ACC and RSC/RSC	AM nucleus sends fibers into posterior cingulate cortex while AV, AD, LD sends fibers BA 29 (RSC).
Horikawa et al.	1988	Fast blue (FB) and Rhodamine microspheres (RH) retrograde tracing	51 Mole rats	ACC and PCC/RSC	ACC and PCC/RSC	AD, AV, LD to adjacent posterior cingulum (A29/P29). AM primarily to anterior cingulum (A24). Rats only have BA 24 and 29, lack other cingulate areas.
Shibata and Naito	2005	PHA-L anterograde and retrograde tracing	37 rats	ACC	ACC	Rostral AM sends fibers to rostral ACC, caudal AM sends fibers to caudal ACC, laminar distribution.
Shah et al.	2012	Dissection	10 humans	ACC	*None described*	Fibers are seen projecting primarily to BA 24, however, it is not possible to disaggregate the thalamocingulate tract from the remainder of the anterior thalamic radiation and rule out connection with ventral cingular areas close to or including BA 23.
Jang and Yeo	2013	Probabilistic fiber tracking using diffusion tensor tractography (DTT)	26 humans	ACC	ACC	Thalamocingulate tract traverses anterior limb of internal capsule to BA 24 to terminate posteriorly through cingulum.
Wei et al.	2017	Seed based diffusion tensor imaging (DTI) tractography	8 humans, 2 tracts failed reconstruction	ACC	PCC/RSC	BA 24 of the ACC set as the region of interest. Alternative hypotheses excluded.


*The cingulate entry row categorizes the findings of each study regarding the entry point into the cingulate cortex. The cingulate termination row categorizes the findings of each study regarding the termination point once enclosed within the cingulate cortex. Notes relating to each study are added to describe either the course of the tract and/or how each thalamic nucleus connects to specific cingular regions. ACC, anterior cingulate cortex; AD, anterodorsal nucleus; ATN, anterior thalamic nucleus; AM, anteromedial nucleus; AV, anteroventral nucleus; BA, brodmann area; LD, laterodorsal nucleus; PCC, posterior cingulate cortex; RSC, retrosplenial cortex.*

### Studies Showing Fiber Entry Into the Anterior Cingulate Cortex

The first group of studies found that the thalamocingulate tract passes dorsally and laterally from the ATN through the anterior limb of the internal capsule before turning rostrally and dorsally to enter the ACC from the subgenual cingulate area to BA 24, entering the cingulum and coursing caudally. Clark and Boggon (1933) confirmed this using lesion-induced cellular degradation. They documented connections from the AV nucleus in the rat and the AV and AD nuclei in the cat to the caudal cingular region. The course of the tract was observed to pass rostrally to the level of the septal region (below the rostrum of the corpus callosum), before turning medially to join the cingulum bundle roughly in the region of the anterior cingulate cortex. Using retrograde tracing techniques, Domesick (1970) similarly described the course of the anterior thalamic fibers in the rat as passing rostrally and entering the cingulum in the anterior region of the cingulate cortex before coursing rostro-caudally once enclosed in the cingulum. Another study utilizing retrograde and anterograde axon tracing of labeled thalamic neurons had findings in accordance with the prior studies and additionally found reciprocal projections from the AM, AV, and AD nuclei to the ACC (Shibata and Naito, 2005). The description of a massive direct projection from each of these anterior thalamic fibers implies agreement with the anterior-only hypothesis for the course of the thalamocingulate tract.

More recent human studies also support this anterior-only hypothesis. The thalamocingulate tract was shown to pass from the ATN through the fibers of the anterior limb of the internal capsule and entering the under-surface of the cingulum in ten human cadaveric brain specimens by Shah et al. (2012). The entry into the cingulate gyrus is not described, however the dissection images demonstrate a massive anterior thalamic radiation projecting anteriorly in a fan-like shape to BA 32, 24, and 23. Jang and Yeo (2013) attempted to isolate these fibers by using diffusion tensor imagining-based probabilistic fiber-tracking to map the course of the thalamocingulate tract. One seed region of interest was placed on the entirety of the cingulate gyrus, while two target regions of interest were placed on the axial aspect of the anterior limb of the internal capsule and on the coronal aspect of the ATN. They show the tracts coursing rostrally after passing the anterior limb of the internal capsule and entering the cingulum bundle in the rostral anterior cingulum BA 32 and 33 before coursing caudally to the posterior cingulum. In Wei et al.’s (2017) study of eight human subjects, deterministic rather than probabilistic fiber tractography was used to visualize the fiber tracts among Papez circuit hubs. Regions of interest were defined as the ATN and the portions of BA 24 corresponding to the ACC as defined by the Automated Anatomical Labeling digital atlas (Tzourio-Mazoyer et al., 2002) and the mid-sagittal plane served as the region of avoidance. The thalamocingulate was visualized in six out of the eight patients and was found to terminate primarily in the RSC and PCC with only few tracts terminating in the BA 24. This analysis did not have the objective of determining the interfacing portion of the cingulate cortex with the thalamocingulate tract, however, the tractography images that they provided show a laminar entry of thalamocingulate fibers emerging from the anterior capsule medially into the anterior to mid-cingulate cortex BA 24 before passing rostrally and terminating in the PCC and RSC. Therefore, this study can be considered to have visualized the tract as having an anterior trajectory. It should be noted that no alternative course could have been visualized given the constraints imposed by the designated regions of interest.

In accordance with the anterior projection hypothesis, Jang and Yeo’s (2013) diffusion tensor imaging (DTI) methodology has been used in three clinical cases to attempt reconstructions of damaged tract and compare them to controls. In the first case the reconstruction of a Papez circuit of a male patient 10 weeks post-hypoxic ischemic traumatic brain injury (TBI) was compared to a healthy control reconstruction, showing a thinning of the right thalamocingulate tract and failure to reconstruct the left thalamocingulate tract (Jang and Kwon, 2016). A second case reconstructed the tracts of a female 3 weeks post-TBI with loss of consciousness and memory impairment and a male 14 months post-TBI. Both patients had normal right thalamocingulate reconstructions, while in the first the left tract was thinner and discontinuous while in the second the left tract failed reconstruction (Yang et al., 2016). In the third case, a female patient with normal pressure hydrocephalus experienced injury to her right anterior thalamus and associated neural tracts as a result of a ventriculoperitoneal shunt. In that case DTI reconstruction of the thalamocingulate tract on the right side was successful and on the left was unsuccessful (Jang and Seo, 2016). All three studies display a massive anterior projection into the anterior cingulate gyrus.

### Studies Demonstrating Fiber Entry Through Both Rostral and Caudal Cingulum Components

Two of the thirteen studies found that at least significant portions of the thalamocingulate tract pass into both rostral and caudal components of the cingulum. Mufson and Pandya (1984) used autoradiographic tracer techniques to study the course and termination of thalamocingulate fibers in the rhesus monkey. They found thalamocingulate fibers emerging from the ATN both rostrally and caudally, occupying the ventral aspect of the cingulum bundle anteriorly near the frontal lobe as well as to BA 23 and 29 of the PCC and RSC, distributed in a fan-like laminar structure. Horikawa et al. (1988) found a similar finding using Fast Blue and Rhodamine microspheres to analyze the projections of the ATN to the cingulum. The AD, AV, and LD nuclei made what were described as direct projections to the adjacent posterior cingulum (BA 29), as rat specimen do not have a structure homologous to BA 23, while the AM nucleus projected directly to the anterior cingulum (BA 24), indicating that the course of the thalamocingulate directly passes into both areas rather than through alternative routes.

### Studies Showing No Determined Pathway Prior to Termination

Despite being cited in the literature for their descriptions of the course of the thalamocingulate tract, some studies provided information regarding the termination without more than implicit reference to its course. This is exemplified by Niimi’s (1978) electrode-induced degradation tracing study that examined anterior thalamic afferents in the cat. The AD nucleus was found to terminate at the RSC, the AV nucleus was found to terminate at the PCC and RSC, and the AM nucleus was found to terminate at the ACC only. This finding may be compatible with both hypothesized courses, either by direct connection to both rostral and caudal regions or by a single anterior course followed by a caudal pathway once enclosed in the cingulum bundle. By contrast Baleydier and Mauguiere (1980), using horseradish peroxidase (HRP) techniques, identified both afferent and efferent thalamocingulate connections between the various anterior thalamic nuclei and regions of the cingulate cortex. The AV, AD, and LD nuclei were found to terminate at BA 23 (ACC), while the AM nucleus was found to terminate at BA 24. Additionally, afferent thalamic fibers from BA 23 projected to the AV, AM, and LD nuclei while the thalamic fibers from BA 24 only projected to the AM nucleus. These findings are compatible with multiple hypotheses regarding cingular region of entry for the thalamocingulate tract.

The remaining studies used horseradish peroxidase (HRP) retrograde and/or anterograde axon tracing in their research. Finch et al. (1984) studied only afferent thalamic fibers to the rat cingulate cortex using HRP retrograde labeling. The AV and AD nucleus was labeled exclusively after injections to the PCC (BA 29), while the AM was labeled only after injections to the ACC (BA 24). Again, the course of the tract was not described, and these findings cannot exclude either hypothesis. Matsuoka’s (1986) HRP study made more granular observations of specific thalamic nuclei in the cat and found afferent projections from the lateral AM and caudomedial part of the AV to the rostral component of the cingulate gyrus, while the medial part of the AM nucleus and the rostromedial part of the AV was related to the caudal component of the cingulate gyrus. Finally, using the same HRP tracing analysis to follow afferent thalamic radiations in the rhesus monkey, Vogt et al. (1987) found that the AM nucleus terminates primarily in BA 23 of the PCC while AV, AD, and LD sends fibers to BA 29 of the RSC. This points to an entirely posterior connection, but again the course of these thalamic afferents was not described.

## Discussion

The 13 research papers examined (Table 1) do not represent a consensus on the anatomy of the thalamocingulate tract. This is due both to their divergent findings of entry into the cingulum (Figure 2) and termination within the cingulum (Figure 3) as well as a result of the lack of validity of comparison of these tracts. Eight studies show the course interfacing the anterior cingulate alone, while two identified the tract entering at both the anterior cingulate as well as the caudal cingular region of the posterior cingulate/retrosplenial gyrus. No studies identified an exclusively caudal cingulate entry. The remaining five studies surprisingly did not specify a course for the thalamocingulate tract, although they are periodically cited as evidence of the course of the tract. Of the studies that described the points of termination of the neurons deep within the cingulum (as opposed to just describing the pathway between the thalamus and the cingulum), two studies found solely anterior cingulate terminations, five found both posterior cingulate/retrosplenial cortex as well as anterior cingulate terminations, and an additional two found purely posterior cingulate/retrosplenial cortex termination fibers from the ATN. The variations in both trajectory and termination fibers from the ATN to the cingulum may relate to differences in connectivity from the constituent anterior thalamic subnuclei (Child and Benarroch, 2013).

### Thalamocingulate Fibers Originating From Subdivisions the Anterior Thalamic Subnuclei

The anterior nuclear group is distinguished from other components of the thalamus by the Y-shaped medullary lamina (Child and Benarroch, 2013). The subunits of the thalamic nuclei each have a different pattern of connectivity and form three different episodic memory subsystems. The anteriomedial nucleus traditionally has been as the only subnucleus of the anterior thalamus with significant reciprocal connections with the anterior cingulate (Jones, 2012), which is supported by five studies which examined individual nuclei in this review while three studies found association with the posterior cingulate. It is thought to be a relay for information from the subiculum, retrosplenial cingulum and mamillary nucleus to the anterior cingulate cortex which in turn projects efferents to the frontal lobe (Wright et al., 2013), an is thought to be associated with executive function (Child and Benarroch, 2013). The anteroventral nucleus by contrast has the most extensive interactions with the subiculum and retrosplenial cingulum and is associated with the promotion of synaptic plasticity in the hippocampal circuit (Wright et al., 2013).

**FIGURE 4:**
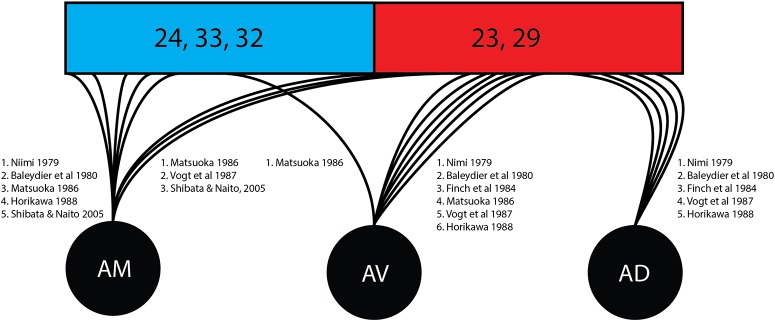
Cingular connectivity of individual thalamic nuclei. Represented is an overview of the quantity of studies tracking the terminations of individual thalamic nuclei once enclose within the cingulate cortex. Brodmann area (BA) 24, 33, and 32 (shown in blue) correspond to the anterior cingulate cortex. Brodmann area (23 and 29 (shown in red) correspond to the posterior cingulate cortex and retrosplenial cortex. AD, anterodorsal nucleus; AM, anteromedial nucleus; ATN, anterior thalamic nucleus; AV, anteroventral nucleus.

As shown in this review (Figure 4) there was divergence in findings of the courses of fibers stemming from specific thalamic nuclei. The anteroventral nucleus was associated with the posterior/retrosplenial cingulate in six studies in this review while associations with the anterior cingulate cortex were found in one study. The anterodorsal nucleus shows connectivity with the mammillary nucleus, postsubiculum, and retrosplenial cortex and has been understood as being involved in the signaling required for special navigation (O‘Mara, 2013). All five studies in this review examining the individual nuclei confirmed connections from the anterodorsal subnucleus to the posterior/retrosplenial cingulate region.

Adjacent to the ATN is the laterodorsal nucleus. The study by Brodel indicated that the laterodorsal nucleus passes fibers to the posterior cingulate/retrosplenial gyrus. By contrast, Shibata and Naito (2005) found that the laterodorsal nucleus projects toward the anterior portion of the cingulate cortex (Shibata and Naito, 2005). Further research may be needed regarding Papez circuit connections, as tracts not originating in the ATN may be misidentified as being part of the thalamocingulate tract. Future studies may require more granular differentiation of specific thalamic nuclei afferents, particularly in diffusion tensor imaging-based studies.

### One, Two or Fanning Thalamocingulate Tracts?

Overall, the argument for the thalamocingulate tract to at least partially enter at the anterior cingulate region is highly convincing, particularly given the consensus among the human studies reviewed. There is also agreement between the studies and original Papez’s, 1937 assertion that the thalamocingulate tract passes through the anterior limb of the internal capsule (as part of the anterior thalamic radiation) that is spatially oriented to interface with the anterior cingulate interface. However, there is also support for a hypothesis that the thalamocingulate tract may also have an extra more caudal entry into the cingulum with two studies directly endorsing this (one in rats and one in primates). However, passage through the anterior thalamic radiation and anterior limb of the internal capsule seems incompatible with a direct trajectory to the more caudal part of the cingulum. If two separate tracts exist, it is possible that studies focusing solely on the anterior thalamic radiation/internal capsule may have missed most posterior traveling fibers. Interestingly, the more recent studies found an solely anterior cingulate interface had study designs that excluded *a priori* the possibility of detecting any potential posterior interface. Therefore, while anterior cingulate connections were confirmed, their findings could neither confirm or exclude a more caudal path of fibers such as those found in X and Y. A more posterior thalamocingulate connection raises intriguing neuroanatomical questions. Is it possible that there are two thalamocingulate tracts – a thalamo-rostral-cingulate and thalamo-caudal-cingulate? Are these distinct tracts that can be identified anatomically or as components of a fan like structure that extends both dorsorostrally and dorsocaudally from the ATN? Are there functional differences between the two systems? Much more research is needed to discover the exact nature of these pathways.

### Common Terminology and Precise Cytoarchitectural Subdivisions Needed

Discontinuous and non-specific descriptive terminology throughout this review suggests the need for both more precise application of terminology for the cingulate cortex as well as the definition of a protocol of nomenclature for the thalamocingulate tract itself. In terms of nomenclature, cytoarchitectural subdivisions along the cingulate gyrus need to encompass the midcingulate cortex (MCC) in future studies rather than remain reliant on the traditional anterior and posterior cytoarchitechtural classifications (Vogt, 2016). The thalamocingulate tract in future investigations should be analyzed in at least two subdivisions, divided by the internal capsule: i.e., from the anterior thalamus and passage through the anterior limb of the internal capsule and from the internal capsule to the cingulate cortex. Further descriptors regarding the termination of the fibers (i.e., in anterior, mid or posterior cingulate) could also be of benefit, dependent on future findings. A study addressing each of these descriptions should allow for valid and precise confirmation or invalidation of each hypothesis.

### Limitations

This focused review has some limitations, not least the paucity of available literature over the last 80 years delineating the path of the thalamocingulate tract. Since Papez’s original paper only thirteen studies have addressed this key component of the circuit. Overall, more research is needed both across species (from rodent to human), as well as in using all techniques (dissection, tracing and DTI studies). Human studies noted significant anatomical variation in track volumes as well as location among subjects (Jang and Yeo, 2013). Given the low number of subjects in many of these studies, this may be a limiting factor contributing to divergent results. Future research may be oriented toward examining the effect of anatomical variability of human subjects to determine whether this is relevant in determining the cause of heterogeneous findings.

Many studies on non-human assume that the structures in rodents, non-human mammals, and humans are homologous. However, the homologies in cingular regions between human and non-human mammals appear to have varying degrees of accuracy. In the cytoarchitecture of the rat, the anterior cingulate regions have homologous regions corresponding to anterior cingulate and retrosplenial areas 24, 33, 32, and 29, but lack a homologous posterior cingulate region corresponding to area 23 and 31 (Vogt and Paxinos, 2014). For example, in Horikawa et al’s study, only areas 24 and 29 were examined as potential termination points, limiting the validity of these studies in drawing conclusions about human subjects. In several of these studies where fibers were found projecting caudally, there was no disaggregation of the fibers passing to either the retrosplenial or posterior cingulate regions from the ATN. These areas have potentially varied functional significance, including in their potential participation in the Papez circuit (Wei et al., 2017), with the posterior cingulate possibly having a role in emotional salience (Brewer et al., 2013) and the retrosplenial cortex having roles in spatial learning (Sugar et al., 2011).

Similarly, establishing directionality of tracts reliably requires morphological information. Authors using horseradish peroxidase and lesion degradation tracing techniques had a capacity to identify distinctions within the tracts connecting the ATN and cingulum, commonly finding that efferent thalamic fibers connect the nucleus and anterior cingulate cortex, while afferent thalamic fibers arrive at the thalamic nuclei from the posterior cingulate regions (Musil and Olson, 1988). However, diffusion-based techniques are unable to distinguish between afferent and efferent tracts. While diffusion tensor imaging is a powerful tool for identifying the gross fiber architecture of large white matter tracts, defining the smaller and more complicated tracts of the limbic system can be hampered by methodological difficulties such as signal to noise concerns, resolution issues, crossing/kissing fibers misidentification (Roddy et al., 2018). Diffusion-based tractography depends on sound neuroanatomical knowledge to infer where the tracts lie amid the complex intervening generated tracts, so the usefulness of these studies as primary evidence for tract pathways may be limited (Mori and Aggarwal, 2014). Indeed, the reason for compiling this review was to properly identify the trajectory of the thalamocingulate tract for the purposes of future diffusion-based research of the Papez circuit.

## Conclusion

To resolve these unreconciled findings surrounding the thalamocingulate tract we suggest the following: (a) Future research should make use of more consistent and current cytoarchitectural subdivisions for the cingulate cortex, and that this more precise terminology be implemented when discussing the thalamocingulate tract; (b) Dividing the tract into pre- and post- anterior capsular components, and known terminations may also aid accurate resolve conflicting descriptions of the tract; (c) Further anatomical detailed staining and microdissections of the thalamocingulate to delineate its precise trajectory in cadaveric humans and clarify the terminal fiber distribution across the cingulate gyrus and cingulum. This can be complimented by animal dissections; and (d) Developments in *in vivo* diffusion imaging such as high angular radial diffusion imaging (HARDI) (Berman et al., 2013), diffusion spectrum imaging (Wedeen et al., 2008), multishell (Rathi et al., 2011) and diffusion kurtosis imaging (Hansen and Jespersen, 2017) as well as improvements in fiber orientation and estimation techniques such as constrained spherical deconvolution (CSD) (Arrigo et al., 2014), super CSD (Tournier et al., 2008) and Q-ball imaging (QBI) (Descoteaux et al., 2007) are allowing ever greater resolution of the microstructural complexity of white matter tracts *in vivo*. Additionally, these enhanced techniques may be complimented by advances in brain parcellation, including the recent development of a probabilistic atlas of the individual human thalamic nuclei (including the anterior thalamus) (Iglesias et al., 2018). Combining both diffusion imaging and brain parcellation advances would allow greater specificity when investigating the diffusion characteristics of the thalamocingulate tracts.

It is unlikely that Papez fully realized how the legacy of his 1937 paper would impact our comprehension of limbic circuits and the extent to which these would drive research into our understanding of the functions of the hippocampus, hypothalamus, and cingulate cortex. The anatomical descriptions of most of the supporting white matter tracts that compose the Papez circuit do not show significant disagreement on their anatomical course, however, the descriptions of thalamocingulate connections between the nuclei of the anterior thalamus and the cingulum have been vague even since Papez’s descriptions. Variance in the pathway appears to be a result of the complexity of the involved structures, the difficulty in discriminating between neural formations and methodological differences between anatomical dissection, neuronal tracing and diffusion studies. This literature review found two conflicting theories of how the anterior thalamic nuclei connect with the cingulum. Upon traversing the anterior limb of the internal capsule, the thalamocingulate tract seems to interface with the anterior cingulate gyrus alone or both the anterior and posterior cingulate/retrosplenial regions. These results are further complicated by conflicting findings regarding the termination of thalamocingulate fibers in these regions. This tract seems to be more than just a simple single projection and may have multiple anatomico-functionally organized projections dependent on the exact subnuclei involved, the fiber terminations in the cingulum and the anatomical trajectory from the anterior thalamus. Future anatomical and diffusion-based research into the functioning of the Papez circuit in emotional and memory will rely on developments in our understanding of this interface.

## Author Contributions

JW prepared the manuscript, responsible for neuroanatomy, and synthesized the data. ER prepared the manuscript and responsible for neuroanatomy. PT and DB prepared the manuscript, responsible for neuroanatomy, and reviewed the data. HG, PM and KL responsible for neuroanatomy and synthesized the data. VO’K reviewed the manuscript and provided the resources. EO’K prepared the manuscript and synthesized the data. DR reviewed the manuscript and designed the study.

## Conflict of Interest Statement

The authors declare that the research was conducted in the absence of any commercial or financial relationships that could be construed as a potential conflict of interest.
